# Pioglitazone Has a Null Association with Inflammatory Bowel Disease in Patients with Type 2 Diabetes Mellitus

**DOI:** 10.3390/ph15121538

**Published:** 2022-12-11

**Authors:** Chin-Hsiao Tseng

**Affiliations:** 1 Department of Internal Medicine, National Taiwan University College of Medicine, Taipei 10051, Taiwan; ccktsh@ms6.hinet.net; 2 Division of Endocrinology and Metabolism, Department of Internal Medicine, National Taiwan University Hospital, Taipei 10002, Taiwan; 3 National Institute of Environmental Health Sciences, Zhunan 35053, Taiwan

**Keywords:** diabetes mellitus, inflammatory bowel disease, pharmacoepidemiology, pioglitazone, Taiwan

## Abstract

Pioglitazone shows potential benefits in inflammatory bowel disease (IBD) in preclinical studies, but its effect in humans has not been researched. We used a nationwide database of Taiwan’s National Health Insurance to investigate whether pioglitazone might affect IBD risk. We enrolled 12,763 ever users and 12,763 never users matched on a propensity score from patients who had a new diagnosis of type 2 diabetes mellitus between 1999 and 2008. The patients were alive on 1 January 2009, and they were followed up for a new diagnosis of IBD until 31 December 2011. Propensity score-weighted hazard ratios were estimated, and the interactions between pioglitazone and major risk factors of IBD (i.e., psoriasis, arthropathies, dorsopathies, chronic obstructive pulmonary disease/tobacco abuse, and any of the above) and metformin were investigated. At the end of the follow-up, 113 ever users and 139 never users were diagnosed with IBD. When compared to never users, the hazard ratio for ever users was 0.809 (95% confidence interval: 0.631–1.037); and none of the hazard ratios for ever users categorized by tertiles of cumulative duration and cumulative dose reached statistical significance. No interactions with major risk factors or metformin were observed. Our findings suggested a null effect of pioglitazone on IBD.

## 1. Introduction

Inflammatory bowel disease (IBD), generally classified as Crohn’s disease (CD) and ulcerative colitis (UC) in clinical practice, is characterized by chronic and relapsing colitis due to excessive expression of various inflammatory mediators. Clinical manifestations include watery diarrhea, fatigue, weight loss, abdominal pain, and bleeding, and sometimes it can be life-threatening [1,2]. It is difficult to differentiate between CD and UC from clinical presentations, and laboratory examinations, including colofibroscope, are necessary for more accurate diagnosis [1]. It has been estimated that 10–15% of the population may have some type of colitis [2]. The prevalence of IBD is approximately 0.3% in North America, Oceania, and Europe, and its incidence is either stable or decreasing in these countries. However, since the 1990s, an increased incidence has been observed in newly industrialized countries in Asia, South America, and Africa [3,4]. In Taiwan, the incidence is increasing, and the annual percentage change has been estimated to be 4% to 5% [3].

The etiology of IBD remains to be investigated, but the interplay among the host, microbiota, and environmental factors is important [2,5,6]. More than 230 genetic loci relating to major histocompatibility complex, pattern recognition, inflammation, and apoptosis have been identified [1,7]. However, environmental risk factors relating to industrialization, sanitation, and hygiene are important, and specific risk factors may include metabolic syndrome, lack of exercise, work shift, dietary patterns (less intake of fiber-containing vegetables, fruit, cereal, and nuts, and more intake of calorically dense diet, elaborate meat, high-fat diet, and high-sugar diet), animal protein, milk formula feeding, vitamin D deficiency, excessive sanitation, psychological stress, history of childhood infection and vaccination and use of oral contraceptives, non-steroidal anti-inflammatory drugs and antibiotics [4,7,8]. On the other hand, breastfeeding may provide protection against IBD [4]. Cigarette smoking and appendectomy both seemed to aggravate CD but might alleviate UC [4]. Metabolites derived from gut microbiota play important roles in mediating the hosts’ immune response and release of inflammatory cytokines and, thus, are pivotal in the development of IBD [9].

Thiazolidinedione (TZD) activates the peroxisome proliferator-activator receptor gamma (PPARγ) and improves insulin resistance and has been used for lowering blood glucose in patients with type 2 diabetes mellitus. In in vitro and in vivo preclinical studies, PPARγ activation has been shown to play a role in the regulation of inflammation and immune response in the colon [10], and the role of PPARγ in the treatment of IBD has long been under investigation [2,5,11,12,13,14,15]. Emodin, a Chinese herb-drug used to treat IBD, may act through its activation of PPARγ-related signaling [16]. A recent study showed that the attenuation of IBD by pioglitazone in cellular and animal studies might act through the prevention of cleaving of annexin A1 in macrophages, leading to reduced secretion of inflammatory cytokines [17]. However, evidence of the use of PPARγ agonists in the treatment of human IBD remains to be explored.

To our knowledge, a population-based study investigating the potential role of PPARγ agonists in the development of IBD in humans is still lacking. In the present study, we aimed to investigate the effect of pioglitazone, a PPARγ agonist in the class of TZD, on the risk of IBD in patients with type 2 diabetes mellitus in Taiwan.

## 2. Results

Table 1 shows the characteristics of never users and ever users in the matched cohort. The two groups were balanced in the distributions of all variables because none of the values of standardized difference was >10%.

Table 2 shows the incidence rates of IBD and the hazard ratios comparing pioglitazone-exposed patients to unexposed patients. The overall hazard ratios and the hazard ratios estimated for each tertile of pioglitazone exposure all favored a null association between pioglitazone use and IBD risk.

The joint effects of and the interactions between pioglitazone and the major risk factors of IBD are shown in Table 3. The joint effect of and the interaction between pioglitazone and metformin are shown in Table 4. All models suggested a null association without any interaction.

## 3. Discussion

### Main Findings

There was a lack of any association between pioglitazone use and IBD (Table 2), and no interaction was observed between pioglitazone and any of the risk factors (Table 3) or pioglitazone and metformin use (Table 4).

a. *Discrepancies with preclinical studies*

Although PPARγ can play a role in the treatment of IBD through the crosstalk between metabolism and inflammation [5] and preclinical studies favor a potential usefulness of pioglitazone in the treatment of IBD [2,5], such a benefit of pioglitazone could not be observed in humans in this observational study. The discrepancies between preclinical in vitro and in vivo studies and this human observational study require some discussion.

First, animal models of colitis are mainly induced by chemicals such as dextran sodium sulfate, trinitrobenzene sulfonic acid, dinitrobenzene sulfonic acid, oxazolone, and intracolonic instillation of acetic acid [2]. It remains to be explored whether colitis induced by these chemicals can completely mimic human IBD. Furthermore, the administered doses of pioglitazone in in vitro and in vivo studies might be much higher than the available concentrations of pioglitazone derived from the clinical doses used for the treatment of hyperglycemia. In Taiwan, the generally accepted maximum dose of pioglitazone is 30 mg, and we rarely use a dosage of up to 45 mg as has been used in Caucasians [18].

Second, the blood concentration of pioglitazone derived from oral administration does not guarantee a sufficient level of pioglitazone to be delivered to the colon for local activation of PPARγ. Recent drug development for the treatment of IBD focuses on more specific delivery of the drugs by using nanotechnology [19] or topically applied PPARγ agonists [20]. Whether these may improve the efficacy of pioglitazone on IBD prevention or treatment are interesting research topics worthy of investigation.

Third, pioglitazone can target multiple organs and tissues, and activation of PPARγ that acts jointly with retinoid X receptor in different types of cells may result in different biological functions, some even counteracting each other. These may explain the various clinical effects observed for different types of cancer and non-cancer diseases in patients treated with pioglitazone. For example, we did observe an improvement in lipid profiles after pioglitazone treatment in a small clinical trial [21] and a significant risk reduction in dementia [22] and chronic obstructive pulmonary disease [23] in observational studies. However, a potentially higher risk of bladder cancer [24] should be attended to in clinical practice.

However, though not statistically significant, the overall hazard ratio of 0.809 (Table 2) favored an approximately 20% risk reduction in association with pioglitazone use. We could not exclude the possibility of lack of power and the confounding by some unmeasured variables such as microbiota and nutrients.

b. *Implications*

At least two clinical implications can be derived from the present study. First, findings observed in preclinical studies suggesting potential usefulness in the treatment of IBD by pioglitazone should never be immediately extrapolated to a clinical implication. At least, our present study did not favor such a benefit of pioglitazone.

Second, pioglitazone does not cause hypoglycemia and shows benefits for cardiovascular diseases [18], especially ischemic stroke [25], and is minimally excreted by the kidney [26]. It should be a candidate drug for glucose lowering in patients who fail their treatment by the first-line drug of metformin, especially when the patients have renal insufficiency or are at a high risk of stroke, dementia, and/or chronic obstructive pulmonary disease. However, pioglitazone should better be avoided in patients with a previous diagnosis of bladder cancer or who are at a high risk of developing bladder cancer, such as a positive family history.

c. *Strengths*

The study has several merits. First, because of the use of a nationwide database of the National Health Insurance (NHI) that has high coverage of >99% of Taiwan’s population, it is reasonable to generalize the findings to the whole population. Second, because we used objective medical records, potential recall bias relating to self-reporting could be avoided. Third, detection biases resulting from different socioeconomic statuses could be minimized because the drug cost-sharing in the NHI is low and can always be waived in veterans, in patients with low income, and in patients who receive prescription refills for chronic disease.

d. *Limitations*

This study also has some limitations. First, we did not have measurement data of some confounders such as anthropometric factors, lifestyle, physical activity, exposure history to some chemicals, history of childhood infection, stress in life, smoking, alcohol drinking, dietary pattern, nutritional status, micronutrient supplementation, family history, and genetic parameters. Second, we did not have biochemical data such as levels of inflammatory cytokines, glucose, insulin, and lipid profiles. Neither did we have indicators of insulin resistance or β-cell function and gut microbiota information for analyses. Third, the outcome of IBD was defined by the International Classification of Diseases, Ninth Revision, Clinical Modification (ICD-9-CM) codes and not by colofibroscopic examinations. Therefore, we could not exclude the potential risk of misdiagnosis in some patients. Because the misclassification was expected to be non-differential, the estimated hazard ratios were supposed to bias toward the null [27,28]. Finally, because the daily dose of pioglitazone used in the Taiwanese and probably also in other Asian populations rarely exceeds 30 mg, whether the clinical use of 45 mg in the Caucasian people [18] would exert a different effect on IBD is an interesting issue that requires additional studies.

## 4. Materials and Methods

### 4.1. The National Health Insurance in Taiwan

Taiwan started to implement the so-called NHI on 1 March 1995. The NHI is compulsory and covers >99.6% of Taiwan’s population. The Bureau of NHI signs contracts with all in-hospitals and more than 93% of all medical settings in Taiwan to provide medical care to the insurants. For reimbursement purposes, computerized medical records, including disease diagnoses, medication prescriptions, and performed procedures, have to be submitted to the Bureau of the NHI. The database can be used for academic research after ethics review and approval. The present study was approved (approval number NHIRD-102-175) by the Research Ethics Committee of the National Health Research Institutes. The readers may refer to our previously published paper for a more detailed description of the database [29].

### 4.2. Enrollment of Study Subjects

Throughout the research period, the ICD-9-CM was used as the disease coding system. Accordingly, diabetes mellitus was coded 250.XX and IBD were coded 555 (regional enteritis) and/or 556 (ulcerative enterocolitis).

**Figure 1:**
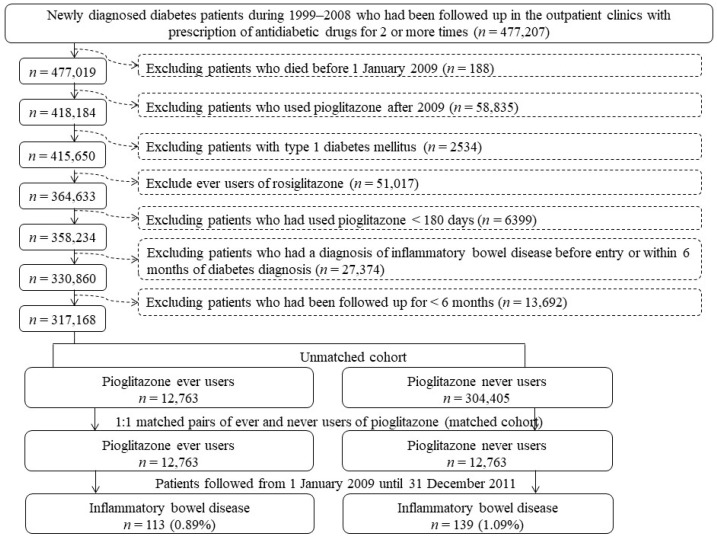
Flowchart showing the stepwise procedures followed in the enrollment of propensity score-matched pairs of pioglitazone ever users and never users.

We created a cohort consisting of propensity score (PS)-matched pairs of ever users and never users of pioglitazone from the NHI database. Figure 1 shows the stepwise procedures. First, we excluded patients whose diagnosis of diabetes mellitus was made during 1995–1998 and then enrolled 477,207 patients who had a first diagnosis of diabetes mellitus made between 1999 and 2008 with a prescription of antidiabetic drugs for at least two times at outpatient clinics. We then excluded step-by-step the following ineligible patients: (1) patients who died before 1 January 2009 (*n* = 188), (2) patients who used pioglitazone for the first time after 2009 (*n* = 58,835), (3) patients who were diagnosed of type 1 diabetes mellitus (*n* = 2534), (4) patients who had ever been treated with rosiglitazone (*n* = 51,017), (5) patients who had used pioglitazone for a short period of <180 days (*n* = 6399), (6) patients who had a diagnosis of IBD before entry or within 6 months of diabetes diagnosis (*n* = 27,374), and (7) patients who had a short follow-up duration of <180 days (*n* = 13,692). As a result, we identified an unmatched cohort consisting of 12,763 ever users and 304,405 never users. We then created PS-matched pairs consisting of 12,763 ever users and 12,763 never users (the matched cohort) based on the Greedy 8→1 digit match algorithm. The PS was created by logistic regression from independent variables that included all characteristics listed in Table 1, as described in more detail previously [29].

We deliberately excluded users of rosiglitazone in the analyses for the following reasons. In Taiwan, only rosiglitazone and pioglitazone in the class of TZD have ever been marketed. In addition to their glucose-lowering effects, these two drugs show different safety profiles in several clinical aspects. For example, the meta-analysis published in 2007 that suggested a potential link between rosiglitazone and myocardial infarction and cardiovascular death [30] has led to the withdrawal of rosiglitazone from the markets or the discontinuation of its use in many countries, including Taiwan. On the contrary, clinical trials suggest that pioglitazone significantly improves lipid profiles [21] and reduces cardiovascular diseases in patients with type 2 diabetes mellitus [18] or in patients with ischemic stroke and insulin resistance [25]. Therefore, in the analyses of the safety profile and the risk association with cancer or other non-cancer diseases, pioglitazone and rosiglitazone should be viewed as two different entities.

### 4.3. Potential Confounders

Potential confounders included in the analyses are listed in Table 1. The occupation was classified as class I (civil servants, teachers, employees of governmental or private businesses, professionals and technicians), class II (people without a specific employer, self-employed people or seamen), class III (farmers or fishermen), and class IV (low-income families supported by social welfare, or veterans). We defined the use of corticosteroids as a consistent use of ≥ 90 days. The ICD-9-CM codes for the disease diagnoses have been reported previously [22].

### 4.4. Statistical Analyses

We used the SAS statistical software version 9.4 (SAS Institute, Cary, NC, USA), as a tool for analyses being conducted in the matched cohort. *p* < 0.05 was considered statistically significant.

We calculated the standardized difference for each covariate and defined a value > 10% as an indicator of potential confounding from the variable, which is generally adopted by many investigators [31].

We calculated two parameters for the assessment of a potential dose-response relationship, i.e., the cumulative duration of pioglitazone therapy (expressed in months) and the cumulative dose of pioglitazone therapy (expressed in mg). The incidence density of IBD was calculated with regard to pioglitazone exposure. The numerator of the incidence was the number of new cases of IBD identified during follow-up, and the denominator was the follow-up duration in person-years. We set the follow-up starting date on 1 January 2009 and ended the follow-up on a date no later than 31 December 2011 when whichever of the following events occurred first: a new diagnosis of IBD, death, or the last reimbursement record. We ended follow-up by the end of 2011 because the concern of a potential risk of bladder cancer associated with pioglitazone was raised in that year [24], which might have led to changes in prescription behavior in the attending physicians and nonadherence to the treatment on the side of the patients.

We estimated hazard ratios and their 95% confidence intervals that compared pioglitazone exposure to non-exposure by applying Cox proportional hazards regression incorporated with the inverse probability of treatment weighting using the PS.

Psoriasis, arthropathies, dorsopathies, and chronic obstructive pulmonary disease/tobacco abuse were viewed as potential risk factors for IBD. To evaluate the joint effects of and the interactions between pioglitazone and these risk factors, we estimated hazard ratios in subgroups categorized by the presence and absence of risk factors and pioglitazone, i.e., (1) risk factor (+)/pioglitazone (−) as the referent group; (2) risk factor (+)/pioglitazone (+); (3) risk factor (−)/pioglitazone (−); and (4) risk factor (−)/pioglitazone (+). The value of P-interaction was also estimated for each model.

Metformin use is associated with a reduced risk of IBD in our previous study [32]. Therefore, we also investigated the joint effect of and interaction between metformin and pioglitazone on the risk of IBD.

## 5. Conclusions

There is a lack of association between pioglitazone use and IBD risk in Taiwanese patients with type 2 diabetes mellitus, and there are no significant interactions between pioglitazone and major risk factors or metformin. Because of the observational design, the potential risk of lack of sufficient power, and the inability to include all potential confounders, further confirmation of our finding is warranted. The daily dose of pioglitazone in Taiwan is generally not more than 30 mg. Whether the use of a higher dose of 45 mg of pioglitazone in Caucasians may exert a clinical benefit on IBD requires further investigation.

## Figures and Tables

**Table 1:** Characteristics of pioglitazone never users and ever users.

Characteristics	Never Users		Ever Users		Standardized Difference
	(*n* = 12,763)		(*n* = 12,763)		
	*n*	%	*n*	%	
**Basic data**					
Age * (years)	61.01	12.17	60.95	11.50	−0.68
Diabetes duration * (years)	6.51	2.74	6.50	2.59	−0.40
Sex (men)	7219	56.56	7218	56.55	−0.03
Occupation					
I	4966	38.91	4920	38.55	
II	2814	22.05	2800	21.94	−0.25
III	2505	19.63	2533	19.85	0.50
IV	2478	19.42	2510	19.67	0.68
Living region					
Taipei	5068	39.71	5097	39.94	
Northern	1326	10.39	1327	10.40	0.10
Central	2044	16.02		15.69	−0.84
Southern	1574	12.33	1609	12.61	0.78
Kao-Ping and Eastern	2751	21.55	2727	21.37	−0.48
**Major comorbidities associated with diabetes mellitus**					
Hypertension	10,370	81.25	10,422	81.66	0.97
Dyslipidemia	10,915	85.52	10,907	85.46	−0.17
Obesity	715	5.60	785	6.15	2.32
**Diabetes-related complications**					
Nephropathy	3290	25.78	3245	25.43	−0.91
Eye disease	4302	33.71	4352	34.10	0.85
Diabetic polyneuropathy	3610	28.28	3617	28.34	0.08
Stroke	3113	24.39	3175	24.88	1.02
Ischemic heart disease	5419	42.46	5479	42.93	0.84
Peripheral arterial disease	3116	24.41	3090	24.21	−0.49
**Factors that might affect exposure/outcome**					
Head injury	436	3.42	440	3.45	0.12
Parkinson’s disease	286	2.24	272	2.13	−0.81
Hypoglycemia	473	3.71	496	3.89	0.85
Chronic obstructive pulmonary disease	5759	45.12	5812	45.54	0.76
Tobacco abuse	486	3.81	504	3.95	0.65
Alcohol-related diagnoses	702	5.50	698	5.47	0.02
Heart failure	1932	15.14	1948	15.26	0.25
Gingival and periodontal diseases	11,232	88.00	11,181	87.60	−1.16
Pneumonia	1576	12.35	1595	12.50	0.29
Pulmonary tuberculosis	388	3.04	444	3.48	2.40
Osteoporosis	2108	16.52	2195	17.20	1.74
Human immunodeficiency virus infection	12	0.09	8	0.06	−1.31
Cancer	1579	12.37	1657	12.98	1.80
Dementia	677	5.30	653	5.12	−0.99
Valvular heart disease	1025	8.03	1043	8.17	0.51
Arthropathies	9628	75.44	9685	75.88	1.02
Psoriasis	419	3.28	374	2.93	−2.19
Dorsopathies	9777	76.60	9792	76.72	0.25
Liver cirrhosis	360	2.82	352	2.76	−0.45
Other chronic non-alcoholic liver diseases	1171	9.17	1210	9.48	0.99
Hepatitis B virus infection	452	3.54	465	3.64	0.52
Hepatitis C virus infection	419	3.28	451	3.53	1.32
Organ transplantation	33	0.26	24	0.19	−1.57
**Antidiabetic drugs and drugs that are commonly prescribed to diabetes patients or drugs that might affect exposure/outcome**					
Insulin	380	2.98	388	3.04	0.43
Sulfonylureas	8849	69.33	8894	69.69	0.58
Metformin	9381	73.50	9389	73.56	0.15
Meglitinide	905	7.09	879	6.89	−1.00
Acarbose	1702	13.34	1742	13.65	0.89
Angiotensin converting enzyme inhibitors/Angiotensin receptor blockers	9404	73.68	9487	74.33	1.38
Calcium channel blockers	7273	56.99	7358	57.65	1.27
Statins	9457	74.10	9505	74.47	0.91
Fibrates	5736	44.94	5728	44.88	−0.15
Aspirin	7594	59.50	7587	59.45	−0.21
Corticosteroids	359	2.81	331	2.59	−1.48

* Age and diabetes duration are shown as mean and standard deviation.

**Table 2:** Incidence rates of inflammatory bowel disease and hazard ratios comparing pioglitazone exposed groups to the unexposed group.

Pioglitazone Use	Incident Case Number	Cases Followed	Person-Years	Incidence Rate (per 100,000 Person-Years)	Hazard Ratio	95% Confidence Interval	*p* Value
Never users	139	12,763	33,988.31	408.96	1.000		
Ever users	113	12,763	34,154.60	330.85	0.809	(0.631–1.037)	0.0937
**Tertiles of cumulative duration of pioglitazone therapy (months)**							
Never users	139	12,763	33,988.31	408.96	1.000		
<11.0	32	4147	10,833.86	295.37	0.727	(0.495–1.068)	0.1044
11.0–19.7	35	4282	11,466.87	305.23	0.745	(0.514–1.079)	0.1191
>19.7	46	4334	11,853.86	388.06	0.942	(0.675–1.315)	0.7253
**Tertiles of cumulative dose of pioglitazone therapy (mg)**							
Never users	139	12,763	33,988.31	408.96	1.000		
<7980	31	4155	10,897.90	284.46	0.700	(0.474–1.033)	0.0726
7980–14,940	37	4266	11,435.47	323.55	0.790	(0.550–1.135)	0.2018
>14,940	45	4342	11,821.23	380.67	0.925	(0.661–1.294)	0.6478

**Table 3:** Joint effects and interactions between pioglitazone and major risk factors of inflammatory bowel disease.

Risk Factor/Pioglitazone Use	Incident Case Number	Cases Followed	Person-Years	Incidence Rate (per 100,000 Person-Years)	Hazard Ratio	95% Confidence Interval	*p* Value
Psoriasis (+)/Pioglitazone (−)	3	419	1124.62	266.76	1.000		
Psoriasis (+)/Pioglitazone (+)	6	374	994.22	603.49	2.329	(0.581–9.332)	0.2325
Psoriasis (−)/Pioglitazone (−)	136	12,344	32,863.69	413.83	1.602	(0.509–5.043)	0.4206
Psoriasis (−)/Pioglitazone (+)	107	12,389	33,160.38	322.67	1.250	(0.396–3.947)	0.7042
*P*-interaction							0.1286
							
Arthropathies (+)/Pioglitazone (−)	117	9628	25,741.21	454.52	1.000		
Arthropathies (+)/Pioglitazone (+)	90	9685	25,978.83	346.44	0.763	(0.580–1.005)	0.0546
Arthropathies (−)/Pioglitazone (−)	22	3135	8247.10	266.76	0.687	(0.427–1.107)	0.1228
Arthropathies (−)/Pioglitazone (+)	23	3078	8175.77	281.32	0.731	(0.457–1.169)	0.1906
*P*-interaction							0.3149
							
Dorsopathies (+)/Pioglitazone (−)	113	9777	26,118.94	432.64	1.000		
Dorsopathies (+)/Pioglitazone (+)	93	9792	26,258.73	354.17	0.820	(0.623–1.079)	0.1558
Dorsopathies (−)/Pioglitazone (−)	26	2986	7869.37	330.39	0.942	(0.603–1.471)	0.7920
Dorsopathies (−)/Pioglitazone (+)	20	2971	7895.87	253.30	0.726	(0.444–1.188)	0.2026
*P*-interaction							0.8522
							
COPD/Tobacco abuse (+)/Pioglitazone (−)	72	5960	15,908.15	452.60	1.000		
COPD/Tobacco abuse (+)/Pioglitazone (+)	56	6038	16,163.84	346.45	0.760	(0.536–1.079)	0.1252
COPD/Tobacco abuse (−)/Pioglitazone (−)	67	6803	18,080.16	370.57	0.860	(0.609–1.215)	0.3932
COPD/Tobacco abuse (−)/Pioglitazone (+)	57	6725	17,990.76	316.83	0.744	(0.520–1.066)	0.1074
*P*-interaction							0.9709
							
Any of the four (+)/Pioglitazone (−)	130	11,333	30,243.27	429.85	1.000		
Any of the four (+)/Pioglitazone (+)	104	11,370	30,483.71	341.17	0.797	(0.615–1.031)	0.0843
All of the four (−)/Pioglitazone (−)	9	1430	3745.04	240.32	0.660	(0.332–1.310)	0.2347
All of the four (−)/Pioglitazone (+)	9	1393	3670.88	245.17	0.668	(0.336–1.327)	0.2492
*P*-interaction							0.6240

COPD: chronic obstructive pulmonary disease.

**Table 4:** Joint effect and interaction between metformin and pioglitazone on inflammatory bowel disease.

Metformin/Pioglitazone Use	Incident Case Number	Cases Followed	Person-Years	Incidence Rate (per 100,000 Person-Years)	Hazard Ratio	95% Confidence Interval	*p* Value
Metformin (−)/Pioglitazone (−)	33	3382	8911.51	370.31	1.000		
Metformin (−)/Pioglitazone (+)	27	3374	8986.75	300.44	0.840	(0.503–1.402)	0.5043
Metformin (+)/Pioglitazone (−)	106	9381	25,076.80	422.70	1.186	(0.793–1.771)	0.4061
Metformin (+)/Pioglitazone (+)	86	9389	25,167.85	341.71	0.950	(0.630–1.433)	0.8074
*P*-interaction							0.6002

## Data Availability

Data contained within the article are not readily available because local regulations restrict the public availability of the dataset to protect privacy. Requests to access the datasets should be directed to C.T., ccktsh@ms6.hinet.net.
